# Use of nicorandil is associated with increased risk of incident atrial fibrillation

**DOI:** 10.18632/aging.204259

**Published:** 2022-09-09

**Authors:** Chien-Chang Lee, Sheng-Nan Chang, Babak Tehrani, Sot Shih-Hung Liu, Chia-Ying Chan, Wan-Ting Hsu, Tzu-Yun Huang, Pang-Shuo Huang, Juey-Jen Hwang, Jien-Jiun Chen, Chia-Ti Tsai

**Affiliations:** 1 Department of Emergency Medicine, National Taiwan University Hospital, Taipei, Taiwan; 2 Center of Intelligent Healthcare, National Taiwan University Hospital, Taipei, Taiwan; 3 Division of Cardiology, Department of Internal Medicine, National Taiwan University Hospital Yunlin Branch, Yunlin, Taiwan; 4 Department of Medicine, Brown University/Rhode Island Hospital, Providence, RI 02912, USA; 5 Department of Family Medicine, National Taiwan University Hospital, Taipei, Taiwan; 6 Department of Epidemiology, Harvard T.H. Chan School of Public Health, Boston, MA 02115, USA; 7 Division of Cardiology, Department of Internal Medicine, National Taiwan University Hospital, Taipei, Taiwan

**Keywords:** atrial fibrillation, nicorandil, translational study

## Abstract

Background: Nicorandil will activate ATP-sensitive potassium channel (KATP). However, activation of potassium channels plays an important role in the mechanism of atrial fibrillation (AF) or atrial flutter (AFL). Whether use of nicorandil might contribute to initiation and/or perpetuation of AF/AFL remained unknown. We determined the relationship between use of nicorandil and risk of atrial fibrillation and determined its molecular mechanism.

Methods: We performed a nested case-control study using a cohort from the National Health Insurance Research Database (NHIRD) of Taiwan. The association between nicorandil use and risk of atrial fibrillation/flutter was estimated by logistic regression model. We also performed molecular, cellular and animal studies to explain the association.

Results: A total of 715 individuals who experienced AF/atrial flutter were matched to 72,215 controls. New use of nicorandil was found to be associated with increased risk for AF/AFL (odds ratio [OR], 2.34; 95% CI 1.07–5.13) compared to nitrate use. We found the expression of KATP subunits Kir6.2 and SUR2A in human and rat left atrial tissues. Furthermore, nicorandil directly shortened action potential duration (APD) in rat left atrium and shortened the QT interval of cultured human induced pluripotent stem cell (iPSC) derived cardiomyocytes (iPSC-CMs).

Conclusions: Use of nicorandil was found to be associated with increased risk of AF/AFL. We also showed the expression of KATP subunits in human atria, and a possible mechanism that use of nicorandil increases the risk of AF through activation of KATP and shortening of atrial APD.

## INTRODUCTION

Atrial fibrillation (AF) is the most common sustained arrhythmia, estimated to affect 1.5–2 percent of the general population in the world [1]. Moreover, the prevalence rate increases with age, up to 9% in people over 80 years old [2]. AF can contribute to serious consequences like hemodynamic impairment and cardioembolic stroke [3]. People having AF are at four to five-fold higher risk of ischemic stroke than those not having AF [4].

Clinical trials and case reports have shown the association between drugs and the induction of AF [4]. Although drug-induced AF may only represent a small proportion of the patients presenting with AF, being an iatrogenic cause, it is important to recognize. However, evidence of the association between drugs and AF is insufficient and mostly according to individual case histories. We do not know if the temporal relationship between drug intake and initiation of AF is an incidental finding or caused by the underlying condition (“confounding by indication). Nicorandil, a combination of nitrate components and sarcolemmal adenosine triphosphate-sensitive potassium channel (KATP) opener, is a potent vasodilator of coronary and peripheral vessels and is used as an antianginal agent. It has been demonstrated that AF is associated with shortening of action potential duration, which involves modified activities of atrial ion currents. An increase in the activity of ATP-sensitive potassium channels via Nicorandil could theoretically reduce atrial action potential duration and could possibly contribute to initiation and/or perpetuation of AF [5].

Nicorandil has been long and commonly used in patients with coronary artery disease. Epidemiologic studies to quantify the relation between nicorandil use and AF have not been performed yet, although AF is a very common arrhythmia with substantial morbidity and potentially serious complications. We aim to perform a nested case-control study in the national health claims database. In addition to the clinical studies that may link nicorandil use to developing AF, we also investigated the possible mechanism by which nicorandil increased the risk of AF using an *in vitro* rat model. Furthermore, human experimental data is the most valuable proof of a new clinical finding, but it is difficult to do an experimental study on a beating human heart. Reprogramming adult somatic cells into induced pluripotent stem cells (iPSCs) has become a powerful model for human diseases. [6] The iPSCs could be further differentiated to beating cardiomyocytes which have a striking similarity of electrophysiological properties with the *in vivo* human heart. [7] Therefore, we also used this iPSC-CM model to test the electrophysiological effect of nicorandil.

## MATERIALS AND METHODS

### Population

We performed a nested case-control study using the National Health Insurance Research Database (NHIRD) of Taiwan. The NHIRD contains records of approximately 1 million people randomly selected from the 23 million beneficiaries of the National Health Insurance of Taiwan. The NHIRD contains complete outpatient and inpatient electronic claim records, individual diagnoses, procedures, and medications prescribed. This study was approved by the institutional review board of the National Taiwan University Hospital, waiving patient written informed consent. This report includes all relevant STROBE elements.

### Study cohort and identification of case patients

Using the NHIRD, we assembled a study cohort that was longitudinally observed from January 2007 through December 2013. The risk of AF/atrial flutter recurrence was not a parameter that we set out to examine; therefore, patients who had been diagnosed with AF/atrial flutter in 2007 and 2008 were excluded for analysis. In addition, patients with a history of cardiotomy or cardiac arrest that can lead to increased risk in AF/atrial flutter were excluded. All cases of AF/atrial flutter identified between since 2009 were incident cases of AF/atrial flutter. To factor in the time-varying risk after initial exposure to nicorandil or nitrate, we excluded all patients who received at least one prescription of nicorandil or nitrate in 2007. The primary case definition used for identification of index cases of AF/atrial flutter was the first occurrence of AF/atrial flutter during the follow-up period with the International Classification of Diseases, Ninth Revision, Clinical Modification (ICD-9-CM) codes of AF (441.1, 441.2, 441.3, 441.4, 441.5, 441.6, 441.7, and 441.9) or atrial flutter (441.0, 441.00, 441.01, 441.02, and 441.03). We included both AF and atrial flutter cases since reported incidences in the literature overlap, and clinical diagnosis and management of both dysrhythmias is similar in clinical practice.

### Population controls

We selected controls by using a risk set sampling scheme. One hundred controls were selected for each case, matched on 5-year age class, sex, and the index date of case diagnosis. Additionally, controls had to be at risk of the event on the case’s index date.

### Medication exposure

Medication exposure was assessed at index date for AF or AFL cases. Use of nicorandil or nitrate was assumed whenever there was any order for a reimbursement code of oral nicorandil with a prescription length of 3 days or longer. The patients received 10–15 mg therapeutic dose in two or three divided doses. To assess the nicorandil use patterns and the risk of AF/atrial flutter, we classified nicorandil usage into 5 patterns. To assess the effect of drug exposure period and risk of AF, we first classified the use pattern into current and past use. Current use refers to the presence of a prescription record of nicorandil within 60 days of index date, past use refers to a record between 61 and 365 days before the index date. To evaluate the effect of starting time of nicorandil use, we classified the use into new use and long-term use. New use refers to nicorandil initiation within 60 days of the index date. Long term use refers to nicorandil initiation between 61 and 365 days before the index date. Lastly, to assess the effect of cumulative days of use on the risk of AF/atrial flutter, we identified patients who had a cumulative prescription length of greater than 120 days. We defined this group of patients as chronic use.

### Covariates

Based on literature reports, [8] we identified 47 covariates in the following categories: demographic, living area, insurance premium level, pre-existing comorbidities, frequency of health care utilization, and co-medications. The variables were summarized in Table 1. We used the ICD-9 codes to compute patients’ CHA2DS2VASc score. To calculate the score, the variables used were age (<65:0 for both sexes, 65–74: +1, ≥75: +2), sex (female ≥65: +1), history of congestive heart failure (+1), hypertension (+1), stroke/TIA/thromboembolism (+2), vascular disease (+1) and diabetes mellitus (+1). Comprehensive details about patient inclusion can be found in the Supplementary Materials and Methods.

**Table 1:** Baseline characteristics of nicorandil, nitrate, and non-users.

	**Non-users (*N* = 71,097)**	**Nicorandil users (*N* = 245)**	**Nitrate users (*N* = 873)**	***P*-value**
**Demographics**				
Gender Male (%)	42,808 (60.21%)	145 (59.18%)	477 (54.64%)	0.0036
Age (Mean ± SD)	69.2 ± 14.35	72.5 ± 9.92	74.68 ± 10.38	<.0001
**Living area**				
Urban Area	34,832 (49.09%)	127 (51.84%)	425 (48.74%)	0.0496
Metro Area	17,655 (24.88%)	62 (25.31%)	190 (21.79%)	
Suburban Area	12,063 (17.00%)	29 (11.84%)	166 (19.04%)	
Countryside Area	6,410 (9.03%)	27 (11.02%)	91 (10.44%)	
**Insurance premium level**				
Dependent	9,334 (13.15%)	13 (5.31%)	97 (11.12%)	<.0001
($1–$19,999)	16,179 (22.8%)	70 (28.57%)	259 (29.7%)	
($20,000–$39,999)	28,102 (39.6%)	115 (46.94%)	378 (43.35%)	
(≥$40,000)	17,345 (24.44%)	47 (19.18%)	138 (15.83%)	
**Pre-existing comorbidities**				
Acute myocardial infarction	124 (0.17%)	13 (5.31%)	34 (3.89%)	<.0001
Congestive heart failure	866 (1.22%)	35 (14.29%)	115 (13.17%)	<.0001
Peripheral vascular disorder	882 (1.24%)	16 (6.53%)	38 (4.35%)	<.0001
Cerebrovascular disease	4,170 (5.87%)	30 (12.24%)	185 (21.19%)	<.0001
Dementia	1,305 (1.84%)	11 (4.49%)	46 (5.27%)	<.0001
Chronic pulmonary disease	2,948 (4.15%)	22 (8.98%)	84 (9.62%)	<.0001
Rheumatologic disease	459 (0.65%)	2 (0.82%)	14 (1.60%)	0.0022
Peptic ulcer disease	5,758 (8.10%)	33 (13.47%)	182 (20.85%)	<.0001
Mild liver disease	4,779 (6.72%)	19 (7.76%)	92 (10.54%)	<.0001
Diabetes without chronic complications	8,893 (12.51%)	75 (30.61%)	297 (34.02%)	<.0001
Diabetes with chronic complications	2,102 (2.96%)	25 (10.20%)	124 (14.20%)	<.0001
Hemiplegia or paraplegia	450 (0.63%)	3 (1.22%)	14 (1.60%)	0.0009
Renal disease	1,638 (2.30%)	20 (8.16%)	111 (12.71%)	<.0001
Any malignancy	2,593 (3.65%)	6 (2.45%)	60 (6.87%)	<.0001
Moderate or severe liver disease	90 (0.13%)	0 (0.00%)	3 (0.34%)	0.1759
Neurologic disorder	1,291 (1.82%)	4 (1.63%)	32 (3.67%)	0.0003
Psychiatric disorder	5,556 (7.81%)	48 (19.59%)	124 (14.20%)	<.0001
Angina	546 (0.77%)	31 (12.65%)	85 (9.74%)	<.0001
Other ischemic heart disease	1,527 (2.15%)	72 (29.39%)	213 (24.40%)	<.0001
Cardiac valve disease	703 (0.99%)	19 (7.76%)	65 (7.45%)	<.0001
Hypertension	19,230 (27.05%)	155 (63.27%)	612 (70.10%)	<.0001
Hyperlipidaemia	9,700 (13.64%)	84 (34.29%)	285 (32.65%)	<.0001
Percutaneous transluminal coronary angioplasty	82 (0.12%)	11 (4.49%)	42 (4.81%)	<.0001
**Health care utilization**				
Number of Outpatient visit	14.18 ± 15.70	27.19 ± 15.84	28.56 ± 18.37	<.0001
Number of Emergency Department visit	0.23 ± 0.77	0.83 ± 1.49	0.85 ± 1.64	<.0001
Number of Hospitalization	0.15 ± 0.64	0.74 ± 1.30	0.75 ± 1.22	<.0001
**Medication use**				
NSAIDs	18,377 (25.85%)	100(40.82%)	351 (40.21%)	<.0001
Aspirins	6,480 (9.11%)	120(48.98%)	384 (43.99%)	<.0001
Systemic corticosteroids	5,797 (8.15%)	34(13.88%)	131 (15.01%)	<.0001
DMARDs	570 (0.80%)	5 (2.04%)	12 (1.37%)	0.0174
Beta-blocker	6,538 (9.20%)	86 (35.10%)	310 (35.51%)	<.0001
ACE-inhibitors/ARB	4,139 (5.82%)	47 (19.18%)	187 (21.42%)	<.0001
Calcium-channel blocker	14,155(19.91%)	131 (53.47%)	523 (59.91%)	<.0001
Statin	6,277 (8.83%)	69 (28.16%)	277 (31.73%)	<.0001
**Scoring differences**				
Charlson comorbidity score (Median, 25–75th)	0 (0–0)	0 (0–1)	0 (0–1)	<.0001
CHA2DS2-VASc Score (Median, 25–75th)	2 (1–3)	3 (2–4)	4 (2–5)	<.0001
**Outcome**				
AF	585 (0.82%)	39 (15.92%)	91 (10.42%)	<.0001

Abbreviation: DMARD, disease-modifying antirheumatic drugs.

### Culture of induced pluripotent stem cell derived cardiomyocytes (iPSC-CMs) and measurement of field electrogram

iPS cells were obtained from the Academia Sinica and cultured on the probe of MED64 microelectrode system (Alpha MED Scientific Inc.) to record spontaneous field electrogram [9]. The detailed method is provided in the Supplementary Materials and Methods.

### Atrial expression of ATP-sensitive potassium channel (KATP)

KATP channels are hetero-octameric complexes of 4 pore-forming Kir6 channel-forming subunits, each associated with one regulatory SUR subunit. Two Kir6-encoding genes, *KCNJ8* (Kir6.1) and *KCNJ11* (Kir6.2), and two SUR genes, ABCC8 (SUR1) and ABCC9 (SUR2) encode mammalian KATP subunits [10]. We detected the expression of Kir6.1, Kir6.2, SUR1 and SUR2 in human and rat left atria by reverse transcription–polymerase chain reaction (RT-PCR) [10]. The detailed method is provided in the Supplementary Materials and Methods. Because it was not easy to obtain human left atrial tissue, the human atrial sample in this study was from only one 76-year-old man receiving mitral valvular surgery due to severe mitral regurgitation. The human study was approved by the ethical committee and Institutional Review Board of the National Taiwan University Hospital (200911002R). The experimental protocol of rat experiment also conformed to the Guide for the Care and Use of Laboratory Animals (NIH Publication No. 85-23, revised 1996) and was approved by the Institutional Animal Care and Use Committee of the National Taiwan University College of Medicine [11].

### Animal model and electrophysiological studies

Wistar rats (300–350 g) received intraperitoneal injection of Zoletil (125 mg tiletamine hydrochloride, 125 mg zolazepam hydrochloride) (20 mg/kg) and ECG and action potentials were recorded. The detailed method is provided in the Supplementary Materials and Methods. In brief, the ECG leads were fixed to the four limbs of the rat to record ECG. The anesthetized rat was then endotracheally intubated and mechanically ventilated. The chest was opened through sternal incision and a stimulating electrode was attached to the right atrium for atrial tachypacing to induce AF. For action potential recording, a glass microelectrode filled with 3M KCl was attached to the left atrium. At the end of the study, the animals were euthanized by cervical dislocation. The experimental protocol conformed to the Guide for the Care and Use of Laboratory Animals (NIH Publication No. 85-23, revised 1996) and was approved by the Institutional Animal Care and Use Committee of the National Taiwan University College of Medicine [11].

### Statistical analysis

**Figure 1:**
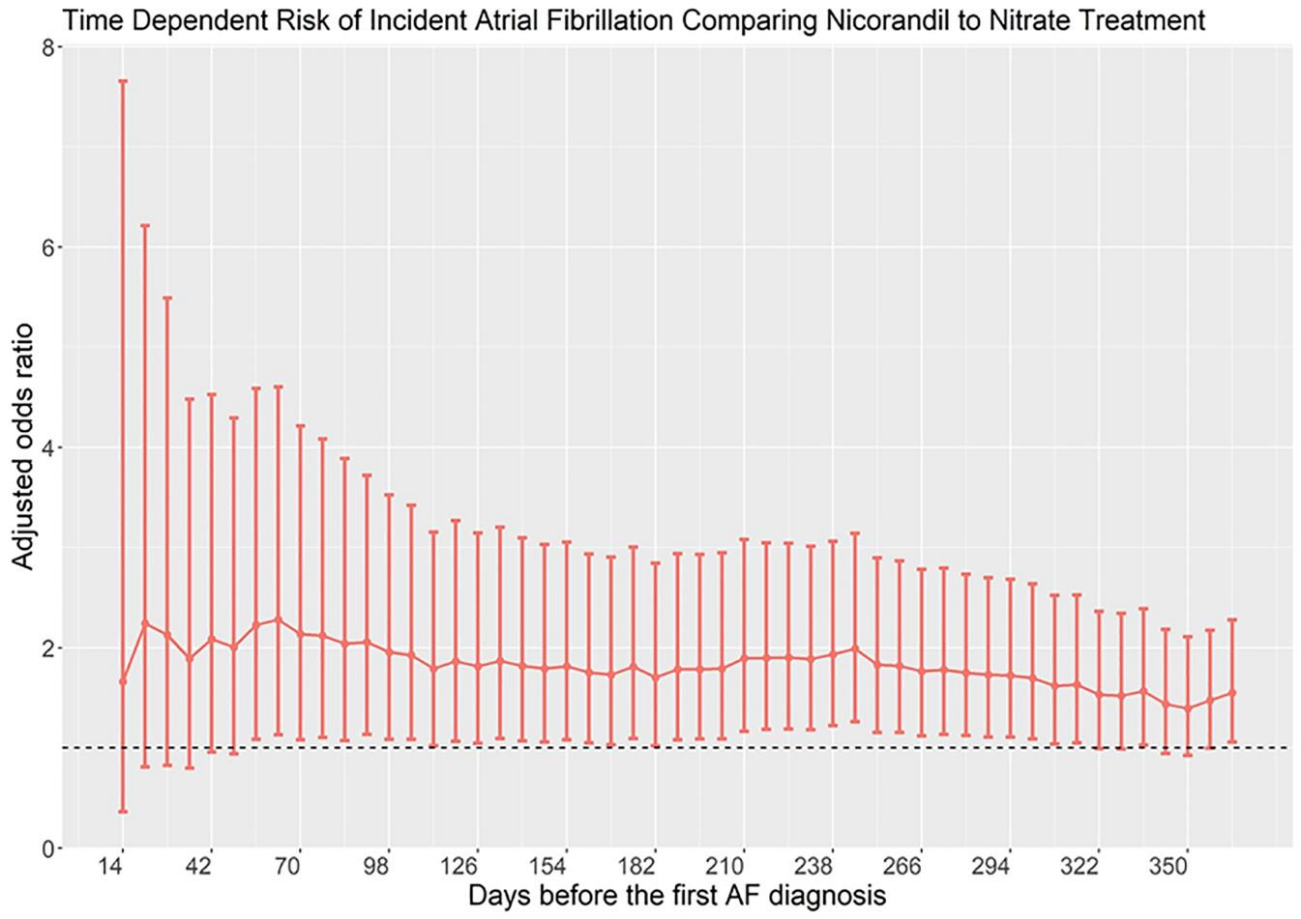
Time-dependent risk of atrial fibrillation in patients taking nicorandil as compared with nitrate.

We used a conditional logistic regression model to compute the odds ratio of AF/atrial flutter, and its 95% confidence interval, associated with use of nicorandil compared to nonuse. Under a time-matched case-control sampling scheme, the odds ratio provided an unbiased estimate of the rate ratio in the cohort. All potential confounders listed in Table 1 were entered into the model for adjustment. To account for the potential unmeasured confounders, we identified nitrate users in the same cohort as an active comparator. We conducted several sensitivity analyses based on different exposure patterns. As the ad hoc categorization of nicorandil use patterns may not reflect the overall risk period, for complete delineation of the timing of nicorandil exposure and associated risk of AF/atrial flutter, and for exploratory analysis of the presence of protopathic bias, we plotted a time-dependent risk graph in Figure 1.

We first constructed a disease risk score (DRS) for matching cases and controls. The DRS was defined as the probability of developing incident AF/atrial flutter in a given year among all members of the cohort unexposed to nicorandil or nitrate as a function of individuals’ covariates ascertained at the beginning of the year. The DRS was estimated by multivariate logistic regression analysis using incident AF/atrial flutter as the dependent variable and all empirical clinical predictors as independent variables. Each case was matched to a control based on the DRS using the greedy matching algorithm. The DRS-matched cohort therefore created a cohort of individuals with similar risk to develop AF/atrial flutter between case and controls, therefore allowing calculation of unbiased risk estimates at different times of exposure.

Finally, to assess effect modification, the estimates were stratified by age (with a cutoff at 70 years), by the presence or absence of a diagnosis of myocardial infarction before the index date, and by the risk of ischemic stroke as measured by CHA2DS2VASc score. We used an E-value as a sensitivity analysis of the unmeasured confounder (https://www.evalue-calculator.com/). The E-value is an analysis method used to quantify the minimum strength of association that an unmeasured confounder must have with both the exposure and outcome, while simultaneously considering the measured covariates, to negate the observed treatment– outcome association [12].

The molecular data was presented as means ± standard deviations. The parameters before and after drug stimulation were compared with Wilcoxon signed rank test. We used SAS v9.4. (Cary, NC, USA) statistical software for all analyses.

## RESULTS

### Patient population

The source population comprised 1,000,000 persons longitudinally followed from 2007 to 2013. We set the study entry date as Jan 1 2009, years 2007 and 2008 were used as a pre-enrollment period. We excluded patients with missing data on demographics, age less than 18, or AF case onset date on December 31, 2013. 715 cases of incident AF/atrial flutter were identified. We then performed a case-control match by matching each case to 100 randomly selected controls by age and sex using the risk-set sampling method. 72215 patients were included for analysis, which were categorized into non-users, nicorandil users, and nitrate users. Supplementary Figure 1 summarized the flowchart of patient inclusion and exclusion process. Supplementary Figure 2 presents the timeline and case-control scheme of the study design.

### Characteristics of study patients

Table 1 compares the baseline characteristics among nicorandil users, nitrate users and non-users. We also compared the characteristics of cases and controls in Supplementary Table 1. Compared to controls, patients who developed AF/atrial flutter have a higher insurance premium level, frequency of healthcare utilization and comorbidity scores. Except for dementia, rheumatologic disease, malignancy and neurologic disorder, cases have higher prevalence of systemic chronic diseases.

### Risk of AF/atrial flutter and use of nicorandil or nitrate

Table 2 shows the age-sex-year-matched and adjusted risks of AF/atrial flutter associated with different types of nicorandil/nitrate uses. Compared to nonuse, current use of nicorandil was associated with an increased risk of AF/atrial flutter (adjusted RR 9.80, 95% CI: 6.56–14.65). The strength of association significantly increased when we restricted patients to those who started to use nicorandil within 60 days (aRR 31.49, 95% CI: 16.80–59.01). For past use (Use only in −61~−365 days), long term use (Use start from −61~−365), or chronic use (cumulative use > 120 days), the association remains significant. Similar to nicorandil use, use of nitrate was found to be associated with increased, albeit attenuated, risk of AF/atrial flutter.

**Table 2:** Crude and adjusted odd ratios for the association between use of nicorandil and risk of incident atrial fibrillation.

	**Incident atrial fibrillation**	
	**Crude RR**	**Confounder-adjusted RR (95% C.I.)**
**Nicorandil vs. nonuse**		
Current use (Use in 60 days)	23.67 (16.59–33.78), *p* < .0001	9.80 (6.56–14.65), *p* < .0001
New use (Use start within 60 days)	49.19 (27.87–86.82), *p* < .0001	31.49 (16.80–59.01), *p* < .0001
Past use (Use only in −61~−365 days)	10.05 (5.66–17.86), *p* < .0001	3.04 (1.60–5.80), *p* = 0.0007
Long term use (Use start from −61~−365)	13.93 (9.70–19.99), *p* < .0001	5.02 (3.32–7.60), *p* < .0001
Chronic use (Cumulative use > 120 days)	12.69 (6.93–23.21), *p* < .0001	2.91 (1.43–5.91), *p* = 0.0032
**Nitrate vs. nonuse**		
Current use (Use in 60 days)	14.66 (11.58–18.56), *p* < .0001	5.90 (4.47–7.78), *p* < .0001
New use (Use start within 60 days)	24.14 (15.61–37.33), *p* < .0001	13.43 (8.27–21.81), *p* < .0001
Past use (Use only in −61~−365 days)	5.76 (4.02–8.25), *p* < .0001	1.96 (1.31–2.93), *p* = 0.0011
Long term use (Use start from −61~−365)	10.04 (8.04–12.55), *p* < .0001	3.80 (2.91–4.97), *p* < .0001
Chronic use (Cumulative use > 120 days)	9.83 (7.06–13.69), *p* < .0001	2.89 (1.97–4.23), *p* < .0001
**Nicorandil vs. nitrate**		
Current use (Use in 60 days)	1.61 (1.07, 2.43), *p* = 0.0214	1.66 (1.06, 2.60), *p* = 0.0270^*^
New use (Use start within 60 days)	2.04 (1.00, 4.15), *p* = 0.0494	2.34 (1.07, 5.13), *p* = 0.0338^*^
Past use (Use only in −61~−365 days)	1.74 (0.90, 3.40), *p* = 0.1015	1.54 (0.74, 3.23), *p* = 0.2498
Long term use (Use start from −61~−365)	1.39 (0.93, 2.08), *p* = 0.1133	1.32 (0.85, 2.06), *p* = 0.2158
Chronic use (Cumulative use > 120 days)	1.29 (0.65, 2.55), *p* = 0.4622	1.01 (0.47, 2.18), *p* = 0.9802

To account for the potential unmeasured confounders that make users of nicorandil or nitrates having a higher risk of AF/atrial flutter (e.g., users of either nicorandil or nitrates have coronary artery disease which may predispose to AF/atrial flutter), we used nitrate as an active comparator and investigated whether use of nicorandil was associated with a higher risk of AF/atrial flutter than use of nitrates.

Compared with nitrate use, current use of nicorandil is associated with a risk for AF/atrial flutter (OR 1.61, 95% CI 1.07–2.43). The risk further increased for new use (OR 2.34, 95% CI: 1.07–5.13). Past use, long term use, and chronic use did not observe an increased risk.

### Subgroup analysis

The subgroup analyses adjusted by DRS showed the risk of AF/atrial flutter associated with nicorandil to be very similar across all subgroups, except age. The risk of AF/atrial flutter associated with nicorandil use was more than 2 times higher (3.61 vs. 1.23; additive interaction *p* = 0.01) in individuals aged less than 65 than in older patients. Related data are shown in Supplementary Table 2.

### E-value

**Figure 2:**
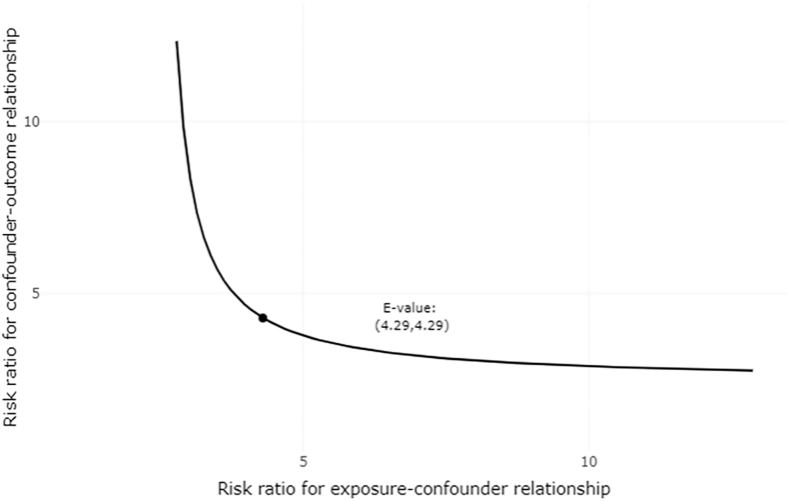
**Plot of sensitivity analysis for unmeasured confounding.** Each point along the curve defines a joint relationship between the two sensitivity parameters that could move the observed association between nicorandil use and incident atrial fibrillation/atrial flutter (RR = 1.66) to the null (RR = 1). Each point along the curve defines a joint relationship between the two sensitivity parameters that could potentially explain away the estimated effect. If one of the two parameters are smaller than the E-value, the other must be larger, as defined by the plotted curve.

The E-value for the association between risk of AF/atrial flutter and current use of nicorandil as compared with nitrate (confounder-adjusted RR = 1.66) was 2.71. Therefore, an unmeasured confounder would only explain away the above association only if it had a relative risk association of 2.71 or greater with both current use of nicorandil and AF/atrial flutter, which is unlikely. The sensitivity analysis plot is shown in Figure 2.

### Basal atrial expression of KATP in the human and rat left atria

**Figure 3:**
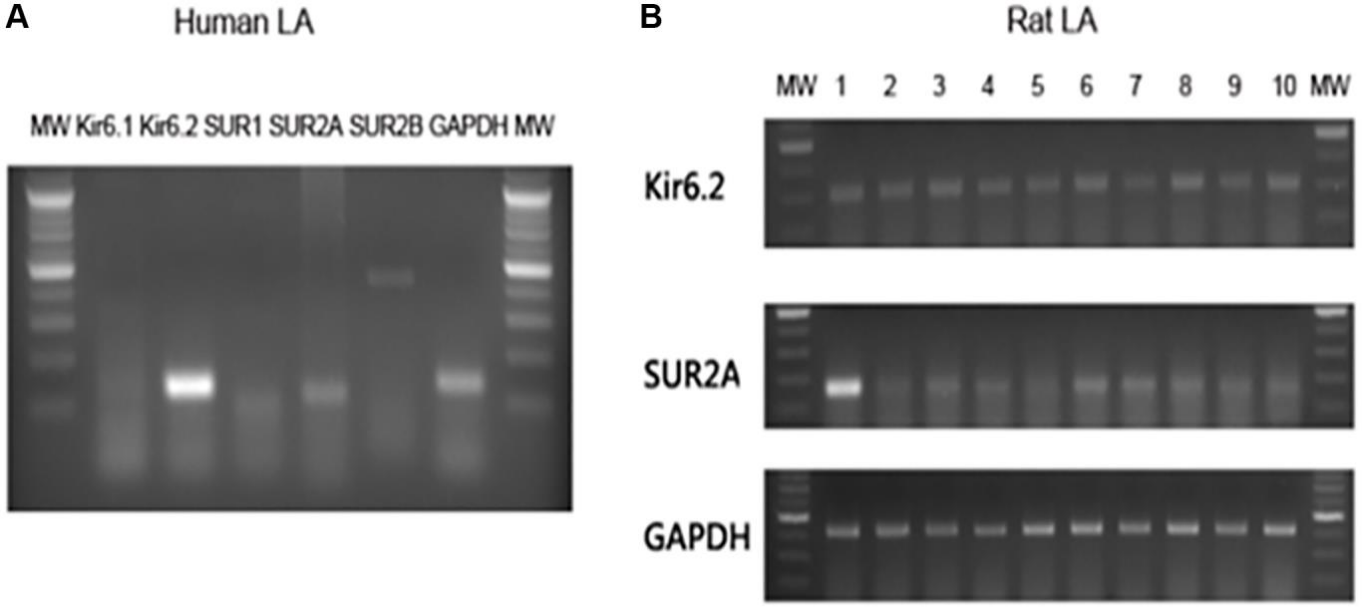
**Basal expressions of KATP subunits in the human and rat atria.** Total ribonucleic acid was isolated and reverse transcription-polymerase chain reaction products with specific primer pairs were visualized by electrophoresis. (**A**) The polymerase chain reaction results of human left atrial tissue. (**B**) The polymerase chain reaction results of 10 rat left atrial samples. Abbreviations: bp: base pair; GP: glyceraldehyde 3-phosphate dehydrogenase; MW: molecular weight maker.

Because the left atrium plays a dominant role in the mechanism of AF, we investigated whether KATP was expressed in the left atrium. Expression of KATP subunits Kir6.1, Kir6.2, SUR1 and SUR2 were detected by RT-PCR. We found the majority of KATP subunits in the human left atrium were Kir6.2 and SUR2A (Figure 3A). Because of limited human left atrial samples, rat left atrial tissue was used for a larger sample size and to perform electrophysiological studies. We also found basal expressions of Kir6.2 and SUR2 subunits in the rat left atria (Figure 3B). Therefore, we subsequently investigated the electrophysiological function of KATP in the rat model.

### Electrophysiological effect of nicorandil on the mammalian atrium

**Figure 4:**
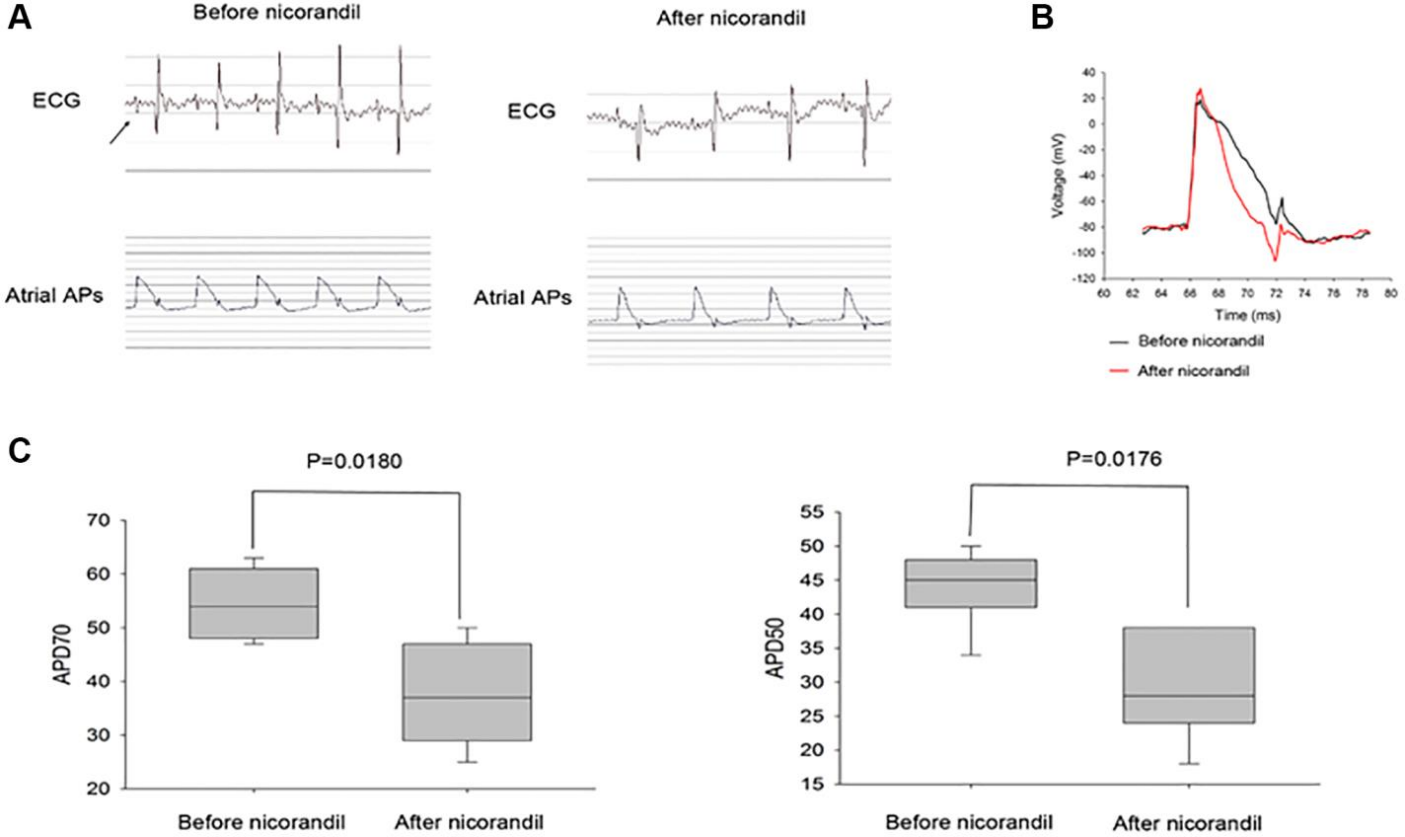
**Nicorandil shortens rat atrial action potential duration.** (**A**) Representative spontaneous rat electrocardiogram (ECG) and atrial action potential duration (APD) tracings of before (left panel) and after (middle panel) nicorandil stimulation (1 mg/kg) are shown. Arrows denote P waves that correspond to atrial APDs. (**B**) Overlap of representative action potentials before (black) and after (red) nicorandil stimulation. (**C**) Summary data of the mean APD50 and APD70 before and after nicorandil stimulation (1 mg/kg) are shown (*n* = 7). The mean APD50 and APD70 are shorter after nicorandil stimulation (1 mg/kg). Data represent mean ± SD; *p* < 0.05 before versus after nicorandil stimulation.

Since there was an expression of major KATP subunits in the rat left atrium, we then sought to investigate the effect of nicorandil on the rat left atrium. We failed to induce sustained AF in normal rats because of the small atrial size. Because shortened action potential duration (APD) is the major surrogate phenotype of AF, we investigated whether nicorandil shortened rat left atrial APD. We found a significant shortening of the rat left atrial APD50 (43.7 ± 5.3 vs. 24.5 ± 7.5, *p* = 0.0176) and APD70 (54.3 ± 6.3 vs. 36.7 ± 9.1 *p* = 0.0180) (Figure 4).

### Nicorandil shortened QT interval of the cultured iPSC-CMs

**Figure 5:**
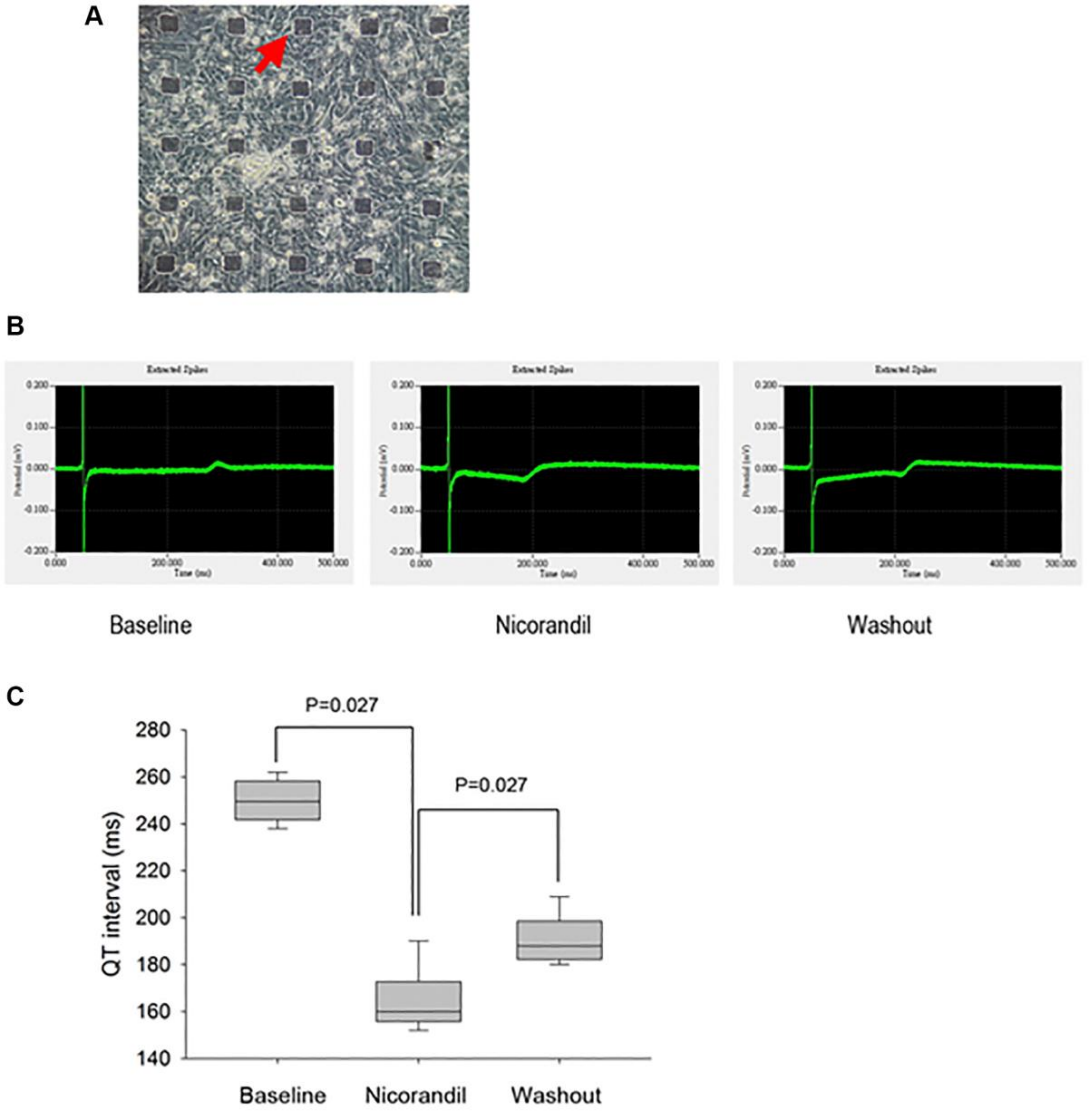
**Generation and analysis of electrograms from human iPSC-CM cultures show nicorandil shortens QT interval of iPSC-CMs.** (**A**) Phase-contrast images show an overview of cell morphology of iPSC-CM cultures on top of the micro electrode array (black rectangular dots; red arrow). (**B**) QT interval in electrogram is a gross phenotype of cellular action potential duration. The electrogram or field potential was recorded from the microelectrodes with iPSC-CMs cultured on top of them. Because the end of the T wave is not prominent, the QT interval is measured as the interval from the Q wave to the peak P wave. Shortening of QT interval is noted after nicorandil (100 uM) stimulation. (**C**) Summary data of QT interval before and after nicorandil stimulation are shown (*n* = 6). Data represent mean ± SD; *p* < 0.05 before versus after nicorandil stimulation.

We had demonstrated that nicorandil shortened atrial APD, which was a surrogate phenotype of AF. However, it is not clear whether nicorandil exerted a similar effect on the human heart. Because it is difficult to do an experimental study on a beating human heart, we tried to use beating iPSC-CMs to simulate the effect of nicorandil on human hearts. Normal beating iPSC-CMs were cultured on the MED64 microelectrode system. The field potential of these beating iPSC-CM monolayers looked very similar to the electrogram of an intact human heart (Figure 4). We failed to directly record APDs of the cultured iPSCs because of the low signal/noise ratio. Since the QT interval of a surface ECG corresponds to the cellular APD, we investigated whether nicorandil shorted QT intervals on this cultured beating iPSC-CM monolayer. We found nicorandil significantly shortened the QT intervals of beating iPSC-CM monolayers. The effect was abolished when nicorandil was washed out (Figures 4 and 5).

## DISCUSSION

We carried out a population-based nested case-control study involving one million national representative participants. Overall, more recent use of nicorandil was associated with higher risk for AF/atrial flutter. New use (use start within 60 days) had the highest risk, followed by current use (use record within 60 days). Past (use only in −61~−365 days), long term (Use start from −61~−365), and chronic (cumulative use >120 days) use were not associated with increased risk of AF/ flutter. These results suggest an acute or subacute effect of nicorandil on the atrial electrophysiology.

Atrial fibrillation is common among the nitrate or nicorandil user population and a non-causal association between nicorandil treatment and AF could arise by confounding because several risk factors for ischemic chest pain and AF are shared. Ischemic heart disease may itself be a risk factor for AF. We used the DRS matching approach to minimize the confounding effect. The DRS matching, like propensity score matching, tries to find 1 (or more) individual(s) with similar DRS in the case and control groups. Following matching, the variation between individuals in risk factors for AF are implicitly controlled.

Several studies have assessed the efficacy and safety of nicorandil, but none have assessed cardiovascular risks associated with nicorandil use. In a previous cohort study, Dunn N and colleagues conducted a prescription-event monitoring (PEM) study of nicorandil to access the safety profile of nicorandil [13]. The study was based on a cohort of 13,260 patients, and ischemic heart diseases were the second most frequently reported events during nicorandil treatment, ranked by incidence density for each event during the first month of treatment per 1000 patient-months. However, many of these events were confounded by indication and may not be causally associated. Some studies have assessed AF risk associated with other anti-anginal medication, including isosorbide mononitrate, atenolol, diltiazem, and verapamil. A narrative review performed to evaluate the quality of evidence associating specific drugs with the occurrence of AF concluded insufficient evidence to support the association [4].

There is biological plausibility that nicorandil may induce AF/atrial flutter. During normal sinus rhythm, time-dependent delayed-rectifier potassium currents and the transient-outward potassium current contribute to action potential repolarization and help to determine action potential duration [14]. Nicorandil exerts two distinct anti-angina mechanisms, acting as both NO donor, and ATP-sensitive potassium channel opener [15]. Its action on ATP-sensitive potassium channels results in shortening of action potential duration, which causes vascular smooth cell hyperpolarization and closure of L-type voltage gated calcium channels and acts to dilate both coronary micro vessels and peripheral resistance arteries [16].

In addition to conducting a clinical study to show nicorandil was associated with an increased risk of incident AF, we also conducted molecular studies to provide a biologically plausible mechanism to explain this clinical finding. We first showed the expression of ATP-sensitive potassium channel in the left atrium, which had never been addressed before. Expression of ATP-sensitive potassium channel in the left atrium raises the possibility that activation of ATP-sensitive potassium channel may augment potassium flow and shorten the atrial action potential duration, subsequently leading to AF [17]. This concept was further enforced by Asano et al. that ATP-sensitive potassium channel might play an important role in electrical remodeling or atrial refractoriness shortening in persistent AF [18]. Interestingly, in the animal model, we found nicorandil directly shortened left atrial action potential duration, which also has been rarely addressed before. We further showed that nicorandil directly shortened the QT interval of cultured human cardiomyocytes. Shortening of action potential duration decreases the wave lengths, thus increases the wavelets of cardiac fibrillation [17].

Given the potential risk of AF/atrial flutter found in this study, the clinical benefit of nicorandil deserves re-evaluation. In a systematic review and meta-analysis with 14 randomized controlled trials enrolling 1947 patients, nicorandil significantly reduced the incidence of myocardial infarction. However, there was no significant reduction of major adverse cerebrovascular and cardiovascular events in nicorandil-treated patients [19]. Another meta-analysis revealed that nicorandil treatment in patients with ischemic heart disease did not reduce revascularization or all-cause mortality [20].

Given the risk of AF associated with nicorandil use, clinicians may need to weigh the benefit against its risk before prescribing nicorandil. Apart from nicorandil, there are other alternative medications for angina. Nitrates, beta-blockers, or calcium channel blockers may be other options. If clinical use of nicorandil is necessary, avoiding prescription to patients aged less than 65 and close monitoring for those whose use starts within 60 days may be imperative to lower the potential risk of nicorandil-associated AF/atrial flutter.

In the present study, we also found the risk of AF associated with nicorandil use was more than 2 times higher in individuals aged less than 65 than in older patients. Usually, old AF patients had more comorbidities such as hypertension or diabetes, and multiple factors, including older age, contribute to AF. Therefore, the contribution of ATP-sensitive potassium channel to AF mechanism may be less in older patients, compared to other comorbidities. On the contrary, there are less comorbidities in younger AF patients, and electrical or ionic remodeling may play a more important role in the mechanism of AF.

There were strengths and weaknesses to the approach we used to detect an increase in the risk of AF/ atrial flutter associated with nicorandil use. Research using the NHIRD of Taiwan has the advantage of its large size with sufficient statistical power for risk analysis. The disease risk score (DRS) matching in a case-control study we used is similar to the propensity score matching in a cohort study that can help minimize confounding. We quantified the potential missing confounders using the e-value approach. The study could not address several uncertainties. First, exposure information was based on nicorandil or nitrate prescriptions and not actual use. However, it is unlikely that cases and controls differed systematically in their adherence to nicorandil or nitrate. Second, although we used all available information on confounding factors and also identified nitrate users as an active comparator for potential unmeasured confounders, we cannot rule out the possibility of residual confounding. Finally, we used a rat model for our electrophysiological studies which might not be a suitable model. The properties and ionic channels of action potential and repolarization of rat are completely different from those of human because of a very high heart rate and very short action potential duration. It is also very difficult to induce AF in normal rats, even under nicorandil stimulation.

## CONCLUSIONS

We found the use of nicorandil, especially new use, is associated with increased risk of AF or atrial flutter. This population-based nested case control study is large in scale and use of nitrate as an active comparator alleviated the concerns of residual confounding. The signal we detected for a possible increased risk during the first few months of nicorandil therapy suggests its acute effect. Our clinical findings might be supported by nicorandil’s acute effect on shortening of atrial action potential duration in animal and cellular studies which might be an indirect evidence of AF promotion.

## Supplementary Materials

Supplementary Materials and Methods

Supplementary Figures

Supplementary Tables
