# Supplementary material for: Use of nicorandil is associated with increased risk of incident atrial fibrillation
Source: Aging (Albany NY). 2022 Sep 9;14(17):6975–92. doi: 10.18632/aging.204259 (PMC9512508; doi:10.18632/aging.204259)
Supplement: Supplementary Materials and Methods [file aging-14-204259-s001.pdf]

SUPPLEMENTARY MATERIALS AND METHODS

Culture of induced pluripotent stem cell derived cardiomyocytes (iPSC-CMs) and measurement of field electrogram

iPS cells were obtained from the Academia Sinica. The cell suspension was transferred to ultra-low attachment flasks (BD Bioscience) and cultured in a 5% C02 atmosphere at 37°C. T25 ultra-low attachment flasks were filled with 5 ml cell suspension, T75 flasks with 15 ml. On day 1 of differentiation, half of the medium was removed and replaced with fresh medium containing 50% mTeSR, 45% DMEM (low glucose), 5% FBS, 1 μMH1152, 100 ng/mL of bFGF and 25 μg/mL of gentamicin. The next day, 2/3 of the medium were replenished with DMEM (low glucose) supplemented with 10% FBS, 50 ng/mL of bFGF and 25 μg/mL of gentamicin. On day 8 of differentiation, the cardiac clusters were every other day with DMEM (low glucose) supplemented with 10% FBS and 25 μg/mL of gentamicin. On day 14 of differentiation, the cardiac aggregates were cultured at 20% O2 and 7% CO2. Between days 9–13 cardiac clusters began to spontaneously beat. Flow cytometry analysis was used to detect the expression of cardiac specific markers between day 16 and 18. The beating cardiomyocytes were then cultured at the probe of MED64 microelectrode system (Alpha MED Scientific Inc., Osaka, Japan) and spontaneous field electrogram was recorded as previously reported [1, 2].

Atrial expression of ATP-sensitive potassium channel (KATP)

KATP channels are heterooctameric complexes of 4 pore-forming Kir6 channel-forming subunits, each associated with one regulatory SUR subunit. Two Kir6-encoding genes, *KCNJ8* (Kir6.1) and *KCNJ11* (Kir6.2), and two SUR genes, ABCC8 (SUR1) and ABCC9 (SUR2) encode mammalian KATP subunits [3]. We detected the expression of Kir6.1, Kir6.2, SUR1 and SUR2 in human and rat left atria by reverse transcription–polymerase chain reaction (RT-PCR). The extraction and quantification of mRNA by means of RT-PCR were performed as previously reported [4–6]. Single-stranded cDNA was amplified with PCR. The PCR products were confirmed by the direct sequencing method. The *glyceraldehyde 3-phosphate dehydrogenase* (GAPDH) gene was used as the internal control for equal loading. The reaction products were subjected to agarose gel electrophoresis. Optic densitometry was performed after the gel was stained with ethidium bromide for semiquantitative measurements of the DNA amount. The SYBR green method was used for quantitative measurement [7]. Expression of mRNA was represented by its ratio to the mRNA of GAPDH. The primers used in the present study were human Kir6.1 forward primer 5′-GGT TTG GAG TCC ACT GTG TGT GTG A-3′ and reverse primer 5′-GGG CAT TCC TCT GTC ATC ATC CTC-3′ (119 bp); human Kir6.2 forward primer 5′-TGA TGA GGA CCA CAG CCT ACT GGA-3′ and reverse primer 5′-AGG ACA GGG AAT CTG GAG AGA TGC T-3′ (125 bp); human SUR1 forward primer 5′-CAA CTG CTG TGT CCA GAT-3′ and reverse primer 5′-ATA CGA ATG GTG ATG TTG GA-3′ (84 bp); human SUR2A forward primer 5′-AAG CAT TCG GTC ATT GTA G-3′ and reverse primer 5′-GCC ACA TAG TAG GTC TGA-3′ (86 bp); human SUR2B forward primer 5′-TGTGATGAAGCGAGGAAATA-3′ and reverse primer 5′-TGACACTTCCATTCCTGAGAGA-3′ (434 bp); human GAPDH forward primer 5′-GTC TCC TCT GAC TTC AAC AGC G-3′ and reverse primer 5′-ACC ACC CTG TTG CTG TAG CCA A-3′ (130 bp); rat Kir6.2 forward primer 5′-CGC ATG GTG ACA GAG GAA TG-3′ and reverse primer 5′-GTG GAG AGG CAC AAC TTC GC-3′ (297 bp); rat SUR2A forward primer 5′-TTG TTC GAA AGA GCA GCA TAC-3′ and reverse primer 5′-GCC CGC ATC CAT AAT AGA GG-3′ (155 bp); rat GAPDH forward primer 5′-TTG CCA TCA ACG ACC CCT TC-3′ and reverse primer 5′-TTG TCA TGG ATG ACC TTG GC-3′ (408 bp).

Animal model and electrophysiological studies

Wistar rats (300–350 g) received intraperitoneal injection of zoletil (20 mg/kg) prior to the electrophysiological studies. The ECG leads were fixed to the four limbs of the animals. The anesthetized mice were endotracheally intubated with a polyethylene tubing through an incision of the trachea and then mechanically ventilated (SAR-830 Small Animal Ventilator, CWE Inc., Ardmore, PA, USA). The chest was opened through sternal incision. Following pericardiotomy, a concentric bipolar stimulating electrode (SNE-100 × 50 mm; RhodesMedical Instruments Inc. Summerland, CA, USA) was fixed to a micro-manipulator (Narishige, Japan) and attached to the right atrium for atrial tachypacing. For action potential tracing a glass microelectrode filled with 3M KCl was attached to the left atrium. Induction of AF was performed by atrial tachypacing at 100 Hz and concomitant short-term asphyxia. All the electrophysiological data were recorded by the IX-214 Data Recorder (iWorx Systems, Inc., Dover, NH, USA) for off-line analysis. At the endo of the study, the animals were euthanized by cervical dislocation. The experimental protocol conforms to the Guide for the Care and Use of Laboratory Animals (NIH Publication

No. 85–23, revised 1996) and was approved by the Institutional Animal Care and Use Committee of the National Taiwan University College of Medicine [7].

Measurement of action potential duration was performed according to our previously reported methods [8]. The activation time was defined as the time point with the maximal dV/dt in the phase zero of the action potential. The repolarization time was defined as the time point with zero second derivative of the dV/dt curve in the phase 3 and phase 4 of the action potential. The APD was defined as the time difference between the activation time and repolarization time.

SUPPLEMENTARY REFERENCES

1. Sala L, Ward-van Oostwaard D, Tertoolen LGJ, Mummery CL, Bellin M. Electrophysiological Analysis of human Pluripotent Stem Cell-derived Cardiomyocytes (hPSC-CMs) Using Multi-electrode Arrays (MEAs). J Vis Exp. 2017; 12:55587. https://doi.org/10.3791/55587 PMID:28570546

2. Tsai CT, Chiang FT, Chen WP, Hwang JJ, Tseng CD, Wu CK, Yu CC, Wang YC, Lai LP, Lin JL. Angiotensin II induces complex fractionated electrogram in a cultured atrial myocyte monolayer mediated by calcium and sodium-calcium exchanger. Cell Calcium. 2011; 49:1–11. https://doi.org/10.1016/j.ceca.2010.10.005 PMID:21168206

3. Nichols CG. Adenosine Triphosphate-Sensitive Potassium Currents in Heart Disease and Cardioprotection. Card Electrophysiol Clin. 2016; 8:323–35. https://doi.org/10.1016/j.ccep.2016.01.005 PMID:27261824

4. Tsai CT, Lai LP, Kuo KT, Hwang JJ, Hsieh CS, Hsu KL, Tseng CD, Tseng YZ, Chiang FT, Lin JL. Angiotensin II activates signal transducer and activators of transcription 3 via Rac1 in atrial myocytes and fibroblasts: implication for the therapeutic effect of statin in atrial structural remodeling. Circulation. 2008; 117:344–55. https://doi.org/10.1161/CIRCULATIONAHA.107.695346 PMID:18172037

5. Tsai CT, Wang DL, Chen WP, Hwang JJ, Hsieh CS, Hsu KL, Tseng CD, Lai LP, Tseng YZ, Chiang FT, Lin JL. Angiotensin II increases expression of alpha1C subunit of L-type calcium channel through a reactive oxygen species and cAMP response element-binding protein-dependent pathway in HL-1 myocytes. Circ Res. 2007; 100:1476–85. https://doi.org/10.1161/01.RES.0000268497.93085.e1 PMID:17463319

6. Lai LP, Su MJ, Lin JL, Lin FY, Tsai CH, Chen YS, Huang SK, Tseng YZ, Lien WP. Down-regulation of L-type calcium channel and sarcoplasmic reticular Ca(2+)-ATPase mRNA in human atrial fibrillation without significant change in the mRNA of ryanodine receptor, calsequestrin and phospholamban: an insight into the mechanism of atrial electrical remodeling. J Am Coll Cardiol. 1999; 33:1231–7. https://doi.org/10.1016/s0735-1097(99)00008-x PMID:10193721

7. Parikh A, Patel D, McTiernan CF, Xiang W, Haney J, Yang L, Lin B, Kaplan AD, Bett GC, Rasmusson RL, Shroff SG, Schwartzman D, Salama G. Relaxin suppresses atrial fibrillation by reversing fibrosis and myocyte hypertrophy and increasing conduction velocity and sodium current in spontaneously hypertensive rat hearts. Circ Res. 2013; 113:313–21. https://doi.org/10.1161/CIRCRESAHA.113.301646 PMID:23748429

8. Tsai CT, Chiang FT, Tseng CD, Yu CC, Wang YC, Lai LP, Hwang JJ, Lin JL. Mechanical stretch of atrial myocyte monolayer decreases sarcoplasmic reticulum calcium adenosine triphosphatase expression and increases susceptibility to repolarization alternans. J Am Coll Cardiol. 2011; 58:2106–15. https://doi.org/10.1016/j.jacc.2011.07.039 PMID:22051334
