# Supplementary material for: Use of nicorandil is associated with increased risk of incident atrial fibrillation
Source: Aging (Albany NY). 2022 Sep 9;14(17):6975–92. doi: 10.18632/aging.204259 (PMC9512508; doi:10.18632/aging.204259)
Supplement: Supplementary Figures [file aging-14-204259-s002.pdf]

## SUPPLEMENTARY FIGURES

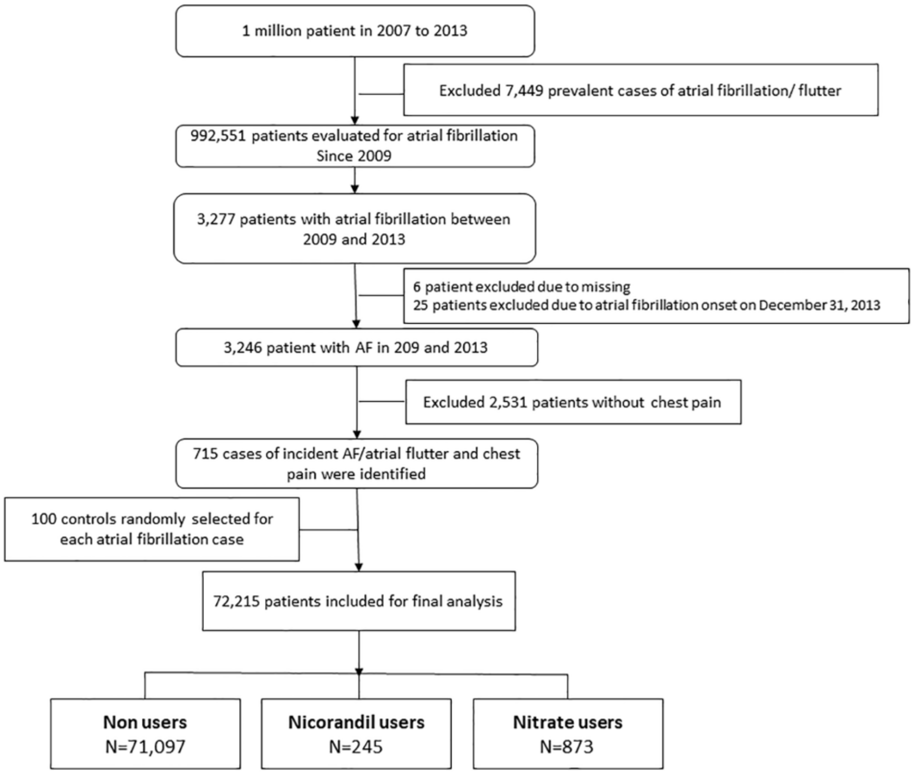


**Supplementary Figure 1. The flowchart of the study design with patient inclusion and exclusion process.**

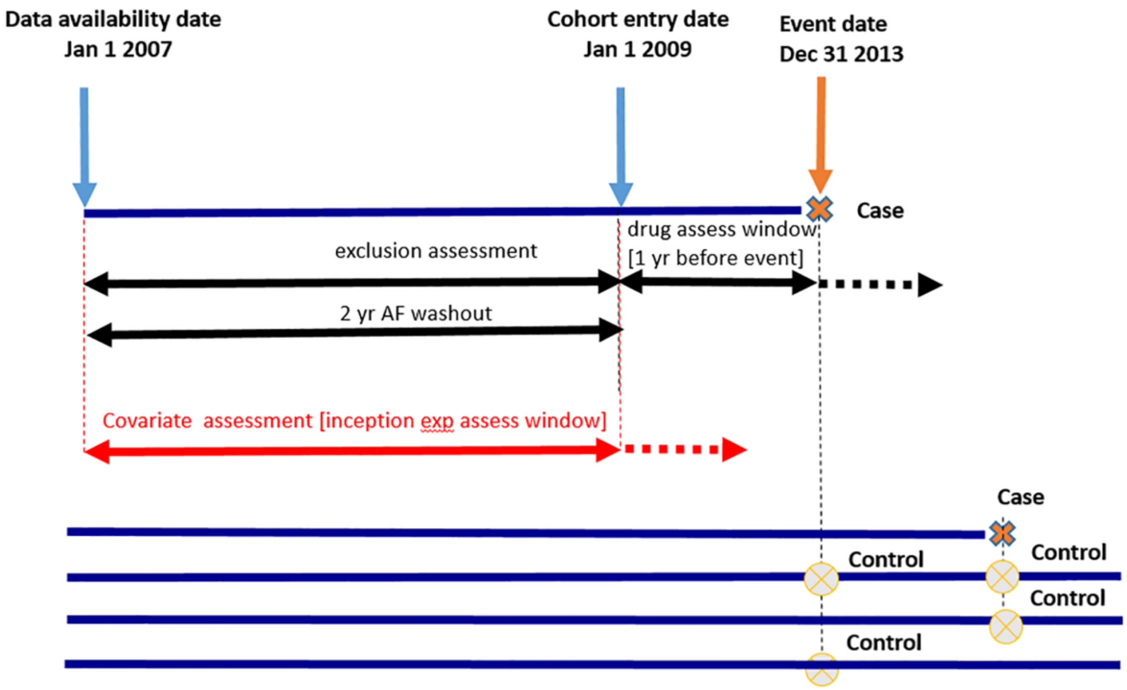


**Supplementary Figure 2. The timeline figure indicates the specific periods used for exclusion of prevalent users, washout cases, identification of cases of controls, assessment of drug exposure and covariates.**
