# Supplementary material for: Use of nicorandil is associated with increased risk of incident atrial fibrillation
Source: Aging (Albany NY). 2022 Sep 9;14(17):6975–92. doi: 10.18632/aging.204259 (PMC9512508; doi:10.18632/aging.204259)
Supplement: Supplementary Tables [file aging-14-204259-s003.pdf]

SUPPLEMENTARY TABLES

Supplementary Table 1. Baseline characteristics of cases and controls.

	Controls (*N* = 71,500)	Cases (*N* = 715)	*P*-value
**Demographics**			
Gender male (%)	43,000 (60.14%)	430 (60.14%)	1.0000
Age (Mean ± SD)	69.28 ± 14.31	68.98 ± 13.53	0.5770
**Living area**			
Urban Area	35,054 (49.12%)	330 (46.15%)	0.0007
Metro Area	17,752 (24.88%)	155 (21.68%)	
Suburban Area	12,118 (16.98%)	140 (19.58%)	
Countryside Area	6,438 (9.02%)	90 (12.59%)	
**Insurance premium level**			
Dependent	9,387 (13.15%)	57 (7.97%)	<.0001
($1-$19,999)	16,348 (22.91%)	160 (22.38%)	
($20,000-$39,999)	28,251 (39.59%)	344 (48.11%)	
(≥ $40,000)	17,376 (24.35%)	154 (21.54%)	
**Pre-existing comorbidities**			
Acute myocardial infarction	163 (0.23%)	8 (1.12%)	<.0001
Congestive heart failure	956 (1.34%)	60 (8.39%)	<.0001
Peripheral vascular disorder	917 (1.28%)	19 (2.66%)	0.0012
Cerebrovascular disease	4,305 (6.02%)	80 (11.19%)	<.0001
Dementia	1,344 (1.88%)	18 (2.52%)	0.2122
Chronic pulmonary disease	2,998 (4.19%)	56 (7.83%)	<.0001
Rheumatologic disease	468 (0.65%)	7 (0.98%)	0.2855
Peptic ulcer disease	5,894 (8.24%)	79 (11.05%)	0.0067
Mild liver disease	4,817 (6.74%)	73 (10.21%)	0.0002
Diabetes without chronic complications	9,114 (12.75%)	151 (21.12%)	<.0001
Diabetes with chronic complications	2,206 (3.09%)	45 (6.29%)	<.0001
Hemiplegia or paraplegia	458 (0.64%)	9 (1.26%)	0.0402
Renal disease	1,709 (2.39%)	60 (8.39%)	<.0001
Any malignancy	2,624 (3.67%)	35 (4.90%)	0.0835
Moderate or severe liver disease	93 (0.13%)	0.00 (0.00%)	0.3346
Neurologic disorder	1,309 (1.83%)	18 (2.52%)	0.1737
Psychiatric disorder	5,649 (7.9%)	79 (11.05%)	0.0019
Angina	6,31 (0.88%)	31 (4.34%)	<.0001
Other ischemic heart disease	1,727 (2.42%)	85 (11.89%)	<.0001
Cardiac valve disease	745 (1.04%)	42 (5.87%)	<.0001
Hypertension	19,637 (27.46%)	360 (50.35%)	<.0001
Hyperlipidaemia	9,917 (13.87%)	152 (21.26%)	<.0001

Percutaneous transluminal coronary angioplasty	125 (0.17%)	10 (1.40%)	<.0001
Health care utilization			
Number of outpatient visit	14.31 ± 15.8	23.03 ± 16.34	<.0001
Number of emergency department visit	0.23 ± 0.78	1.05 ± 1.53	<.0001
Number of hospitalizations	0.15 ± 0.64	1.07 ± 1.28	<.0001
**Medication use**			
NSAIDs	18,572 (25.97%)	256 (35.8%)	<.0001
Aspirins	6,802 (9.51%)	182 (25.45%)	<.0001
Systemic corticosteroids	5,884 (8.23%)	78 (10.91%)	0.0096
DMARDs	578 (0.81%)	9 (1.26%)	0.182
Beta-blocker	6,761 (9.46%)	173 (24.20%)	<.0001
ACE-inhibitors/ARB	4,274 (5.98%)	99 (13.85%)	<.0001
Calcium-channel blocker	14,527 (20.32%)	282 (39.44%)	<.0001
Statin	6,514 (9.11%)	109 (15.24%)	<.0001
Nicorandil	206 (0.29%)	39 (5.45%)	<.0001
Nitrate	782 (1.09%)	91 (12.73%)	<.0001
**Scoring differences**			
Charlson comorbidity score (Median, 25–75th)	0 (0–0)	0 (0–1)	<.0001
CHA2DS2-VASc Score (Median, 25–75th)	2 (1–3)	2 (1–4)	<.0001

Abbreviation: DMARD: disease-modifying antirheumatic drugs.

Supplementary Table 2. The risks of AF in different risk groups.

	Nicorandil vs. Nitrate users risk ratio (95% C.I.)	Additive Interaction *P*-value
**Age**		
Age > 65 years	1.23 (0.71, 2.13)	
Age ≤ 65 years	3.61 (1.58, 8.22)	0.01
**Gender**		
Male	1.37 (0.77, 2.45)	
Female	2.28 (1.12, 4.67)	0.73
**Cardiovascular disease (MI, CHF, PVD, CVA)**		
Presence	1.28 (0.58, 2.83)	
Absence	1.71 (1.10, 2.68)	0.58
**Severity**		
CHA2DS2-VASc 0-2	2.63 (1.29, 5.37)	
CHA2DS2-VASc 0-2	3.49 (1.13, 10.75)	0.97
CHA2DS2-VASc 6-10	3.49 (1.13, 10.75)	
